# Determinants of aggressive behavior: Interactive effects of emotional regulation and inhibitory control

**DOI:** 10.1371/journal.pone.0175651

**Published:** 2017-04-11

**Authors:** I-Ju Hsieh, Yung Y. Chen

**Affiliations:** Institute of Cognitive Neuroscience, National Central University, Taoyuan City, Taiwan; University of Granada, SPAIN

## Abstract

Aggressive behavior can be defined as any behavior intended to hurt another person, and it is associated with many individual and social factors. This study examined the relationship between emotional regulation and inhibitory control in predicting aggressive behavior. Seventy-eight participants (40 males) completed self-report measures (Negative Mood Regulation Scale and Buss-Perry Aggression Questionnaire), a stop signal task, and engaged in a modified version of Taylor Aggression Paradigm (TAP) exercise, in which the outcome was used as a measure of direct physical aggression. We used a hierarchical, mixed-model multiple regression analysis test to examine the effects of emotion regulation and inhibitory control on physical reactive aggression. Results indicated an interaction between emotion regulation and inhibitory control on aggression. For participants with low inhibitory control only, there was a significant difference between high and low emotion regulation on aggression, such that low emotion regulation participants registered higher aggression than high emotion regulation participants. This difference was not found among participants with high inhibitory control. These results have implications for refining and targeting training and rehabilitation programs aimed at reducing aggressive behavior.

## Introduction

Regardless of culture or geographical location, various manifestations of aggression and forms of aggressive behavior may be found among groups of individual. Aggressive behavior has been defined as any behavior intended to harm or injure another organism which is motivated to avoid such treatment [1]. This common yet destructive behavior may be the cause of financial loss, emotional distress, physical injury, or even death, to those exposed to it, not to mention other more indirect interpersonal and social costs to individuals and social groups associated with the victims. Therefore, much effort has been devoted to the understanding and prediction of aggressive behavior.

Previous research has proposed several types of aggressive behavior. The mostly used categories are physical, verbal, and indirect aggression [2]. As each category’s name indicates, physical aggression involves physical harm to others, verbal aggression includes the use of language to hurt people, and indirect aggression involves social manipulation, such as social exclusion that ultimately results in harm to others. Other types of aggression include reactive and instrumental aggression. Reactive aggression (sometimes also referred to as emotional aggression) is a response to provocation or threat, while instrumental aggression (sometimes referred to as proactive aggression) refers to using aggression as means to an end. In the current study, the focus would be on physical reactive aggression.

Previous research has shown that physical aggression can be affected by many factors, such as gender (e.g.,[3, 4]), alcohol use (e.g., [5]), exposure to violent media (e.g., [6]). Several theories have been developed in order to integrate these factors and explain their relationships with one another. Finkel [7] proposed a model (I^3^ theory) aimed at explaining aggressive behavior, taking into consideration many of the known factors that were associated with aggressive behavior. According to the theory, the commission of aggressive behavior can be determinate by the interactions among instigating trigger, impelling forces, and inhibiting force. Instigating trigger is defined as situational events or circumstances with the potential to lower the threshold of carrying out aggressive acts. Instigating factors would increase the likelihood of aggression. Impelling force refers to personality traits or dispositional factors that would increase the likelihood of aggression, particularly when individuals with these traits are confronted with instigating triggers. Finally, inhibiting force is one that would override an aggressive urge. Together, instigating trigger and impelling force would strengthen an aggressive impulse. Inhibiting force, on the other hand, would determine the threshold above which aggressive impulses would transform into actual aggressive behaviors.

Individual components in the I^3^ theory have indeed been found to be associated with aggressive behavior in previous research. For example, instigating triggers, such as provocation [8], social rejection [9], and unpleasant temperature [10], have been found to be related to higher incidents of aggressive behavior and increased aggression. Impelling force such as high impulsivity [11, 12] and trait aggressiveness [13] were also found to predict aggressive behavior. Finally, inhibiting force, such as high self-control [14, 15] and better executive functioning [16, 17] have been found to be inversely associated with incidents of aggressive behavior. However, fewer studies have examined the potential interactions among the three factors in predicting aggressive behavior. One previous study reported an interaction between provocation and self-regulatory process, such that participants with self-regulatory failure allowed their partners to maintain in painful yoga pose for longer periods of time, but only when they were provoked [18]. However, the simplicity of I3 theory’s also presents limitations in understanding the multidimensional nature of the constructs that contribute to a complex behavior such as aggression. For example, it would be challenging to fit the theory into the context of instrumental aggression, where someone would use aggression to get to an ultimate goal. Alternative models have raised possibility of potential interactive effects among predictors of aggression (e.g., [19]).

The current study examined the effects of two inhibiting forces, operationalized as emotion regulation and inhibitory control, in predicting physical reactive aggression. The link between negative emotions and aggressive behavior has been extensively researched. Emotions such as anger, fear, and other generally negative affect have been found to be associated with aggression [20]. Therefore, the ability to regulate negative emotions can be, and has been found to reduce aggressive behavior [21]. Emotion regulation is the ability to modulate ones emotional experience and/or responses [22]. Among the many approaches to examine emotion regulation, one approach is the distinction between top-sown vs. bottom up strategies. Efforts aimed at reframing or reinterpreting emotion provoking stimuli, such as cognitive reappraisal, would involve the top-down (prefrontal areas) modulation of emotion generating brain regions, such as the amygdala [23]. Bottom-up strategies, such as exposure or conditioning, would directly affect the emotion generating brain regions, with less degrees of involvement from the higher brain regions [24]. The current study sought to investigate possible effects of top-down emotion regulation on inhibitory control in predicting aggression.

Inhibitory control is the ability to suppress a strong tendency to act, and is an important component of suppressing aggressive behavior. Raaijmakers, Smidts [25] found that children who showed higher levels of aggressive behavior were significantly more impaired in inhibition than children who showed lower levels of aggressive behavior. Though inhibitory control, usually examined in a motor or behavioral response prevention context, is thought to involve the brain’s prefrontal network, the “top” brain region’s involvement has been found to vary between inhibitory control and other relatively more cognitive tasks, such as dynamic decision making and response selection [26, 27]. In addition, top-down emotion regulation strategies, such as cognitive appraisal, has been found to be associated with the ability to recruit various appropriate brain regions [28]. While both emotion regulation and inhibitory control may be critical components in predicting aggression, they may interact in the process leading up to the execution of aggressive behavior.

One purpose of the current study was to investigate the potential interaction between emotion regulation and inhibitory control in predicting aggressive behavior. Specifically, we examined emotion regulation as a moderating factor for the effects of inhibitory control on aggression. We expected that when provoked, participants high on both emotion regulation and inhibitory control would show the least levels of aggression, while those low on both would show the highest. Due to the relatively more cognitive or top-down nature of emotion regulation, we also expected that it might help reduce aggressive behavior in participants with low inhibitory control.

Another purpose of the current study was to examine aggressive behavior and relationships among the factors in the I^3^ model in an Asian sample. Although much of the previous research on aggression had been conducted in western cultures, the norms for aggressive behavior have been found to vary across cultures. For example, a study by Crystal, Chen [29] compared 11-grade students in three countries: the U.S., Japan, and Taiwan. In this study, Asian students reported less aggression than American students, but Taiwanese students reported more aggression than Japanese students. Another study showed that compared with Chinese or Polish college students, American students reported the highest levels of aggression, followed by Polish and Chinese students [30]. Some have suggested that the differences in levels of aggression among various cultures may be even greater than gender differences in aggressive behavior [31]. This study aimed to examine aggressive behavior and its contributing factors in a Taiwanese sample. As such, we expected that a Taiwanese sample might require a higher provocation level before aggression is shown.

## Methods

### Participants

Participants were 80 undergraduate college students who completed the study for monetary compensation or course extra credit. The study was approved by the Research Ethics Committee at the Research Ethics Office of National Taiwan University. In examining the data for normal distribution, we identified two outliers for NMR based on interquartile rang (IQR), which was computed from Turkey’s hinges. NMR scores lower than 1.5 IQR’s but less than 3 IQR’s from end of the boxplot were labeled as outliers. After removing the two outliers, the sample consisted of 41 men and 39 women. Their mean age was 21.57 (*SD* = 1.75).

### Procedure

Participants’ initial sessions were conducted in a private room, where they were given an introduction to the study and guided through the process and content of informed consent. Participants were assured that all of their questionnaire responses would be kept confidential. After participants signed the consent form, they were asked to complete study questionnaires. Then participants performed a stop signal task and Taylor aggression paradigm in a counterbalanced order. At the completion of the study, the experimenter debriefed the participants and thanked them for their participation.

### Measures

*Aggressive behavior* was measured using the modified version of Taylor aggression paradigm [3]. Participants were told that they were going to play a competitive reaction time game with an unknown opponent, and that whoever was slower would receive a high-pitched noise through the headphone, as punishment. Unknown to the participants at that time, however, was that their opponents were virtual, and their wins/losses were predetermined. At the beginning of each trial, participants would select the level of punishment that their opponents would receive, if their opponents were to lose. The noise setting buttons were labeled on the keyboard from 1 (corresponding to 55 dB) to 10 (corresponding to100 dB). After each trial, participants were informed of the outcome of the trial (i.e., “you won” or “you lost”). Then, participants were shown the punishment level that their opponents had assigned them for that particular trial. For participants who have lost, they would then be exposed to the punishment noise, corresponding to the level that their opponents assigned them.

The frequency of wins and losses (set at 50% for the current study) and the intensities of noise received were predetermined. Participants competed in 96 reaction time (RT) trials, which were divided into three blocks. The average intensity of noise was 2.5 for block one, 5.5 for block two, and 8.5 for block three. Aggressive behavior was measured by the average levels of punishment participants assigned to their opponents for each block.

*Emotional regulation* was measured using the Negative Mood Regulation (NMR) Scale [32]. The NMR is a 30-item scale scored on a 5-point Likert-type scale: 1 (*strong disagree*) to 5 (*strong agree*). The NMR is used to assess one’s ability to relieve or terminate bad moods and emotional distress. Higher score on the NMR would indicate better negative mood regulation. In the current sample, Cronbach’s alpha (*α*) = 0.85.

*Inhibitory control* was measured using stop signal reaction time (SSRT) in a stop signal task [33]. The task included two conditions, a “go” condition and a “stop” condition. The go condition required participants to respond to a visual go signal on the computer screen, by pressing on a designated button on the keyboard. The stop condition involved an audible stop signal, which was presented after the visual go signal, and required participants to inhibit their intention to respond to the go signal. Stop condition occurred on 25% of the trials. Inhibitory control was measured by the latency of the response to stop signal (SSRT). Longer SSRT would indicate worse inhibitory control.

*Trait aggression* was included as a controlling variable, and was measured using the Buss-Perry Aggression Questionnaire, BPAQ [34]. The BPAQ is a 29-item inventory scored on a 5-point Likert-type scale: 1 (*extremely uncharacteristic of me*) to 5 (*extremely characteristic of me*). Higher scores on the BPAQ would indicate higher trait aggression. In the current sample, Cronbach’s alpha (*α*) = 0.85.

*Provocation* was assessed at the end of the experiment, by asking the participants how much they felt “provoked” by their opponents during the experiment.

*Demographic* information was also collected from the participants, which included their age and gender.

### Statistical analysis

Using the SPSS 18.0 software package (IBM, Armonk, NY, USA), we employed one repeated-measures Analysis of Variance Analysis test (ANOVA) to check the effect of provocation. Then we used a hierarchical, mixed-model multiple regression analysis test to examine the effects of emotion regulation and inhibitory control on physical reactive aggression. In Step 1, we included provocation, age, gender, and trait aggression as controlling variables. In Step 2, we included negative mood regulation (NMR) and inhibitory control (SSRT) as predictors. Finally, in Step 3 we added the two-way interaction between emotion regulation (NMR) and inhibitory control (SSRT) as predictors.

## Results

### Preliminary analysis

The mean score for NMR was 103.03 (SD = 12.06), for BPAQ was 75.74 (SD = 13.88), and for SSRT was 219.57ms (SD = 25.95). No significant correlations were found among main study variables, as showed in Table 1.

**Table 1:** Descriptive results and correlation matrix between predictors.

Variable	Mean	SD	1	2	3	4
1. Age (year)	21.57	1.75	1	-.17	-.18	-.17
2. Emotion regulation	103.03	12.06		1	-.16	.13
3. Trait aggression	75.74	13.88			1	-.23
4. Inhibitory control (ms)	219.57	25.95				1

A repeated-measures Analysis of Variance Analysis (ANOVA) was conducted to test main effect of block on the punishment levels participants assigned to their opponents. Results showed a significant main effect of block on assignment of punishment (*p* = .000). Participants’ average punishment (i.e., aggression) increased significantly from Blocks 1 to 3. Post-hoc test (LSD) revealed a significant increase from Block 1 (B1, M = 3.03) to Block 2 (B2, M = 4.63, *p* < .001), and from B2 to Block 3 (B2, M = 6.55, *p* < .001).

As expected, there was a greater increase in punishment level from B2 to B3, than from B1 to B2, indicating higher provocation level in the current sample, possibly due to participants being provoked by greater noise levels received from their virtual opponents [8]. Therefore, the difference in punishment assignment from B2 to B3 was calculated and used as an indicator of aggressive behavior in the current study.

### Main effects analysis

Effects of main study variables on aggressive behavior were then examined using hierarchical, mixed-model multiple regression analysis. Step 1 of the Model included controlling variables of provocation, age, gender, and trait aggression. Step 2 included main study variables of negative mood regulation and inhibitory control. Results indicated no significant main effects for any of the main study variable. Further analysis involving hierarchical entry of the predictors indicated that main effects described above were unaltered by the sequence in which they were entered into the model.

### Moderation analyses

**Fig 1:**
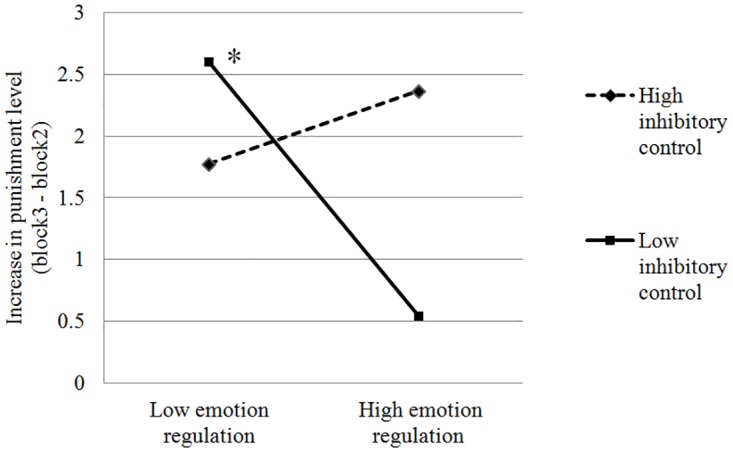
Interactive effect of emotion regulation and inhibitory control on increase in punishment level. a. Predicted values of increase in punishment are plotted 1 standard deviation above and below the mean for NMR [35]. b. The slope for high SSRT (low inhibitory control) is significantly different from zero (*p* = .002), and the slope for low SSRT (high inhibitory control) is not, (*p* > .33).

Next, in Step 3 we added the two-way interaction between emotion regulation (NMR) and inhibitory control (SSRT) in predicting aggressive behavior. Step 3 significantly improved the main effects model, R^2^ = .309, *p* < .02, reflecting a significant NMR x SSRT interaction (Fig 1). For participants with high inhibitory control (low SSRT), there was no significant difference between those high vs. low on NMR scores in predicting aggressive behavior. However, for participants with low inhibitory control (high SSRT), those with higher scores on NMR showed less aggressive behavior than those with lower scores on NMR. In addition, no other significant interactions were found among variables included in Steps 1 and 2 in predicting aggression (ps > .40). We also examined these interactive effects for increases in punishment between Blocks 1 and 2, but did not find any significant interactions.

## Discussion

Result from the current study showed an interactive effect of emotional regulation and inhibitory control in predicting aggressive behavior. For individuals with low inhibitory control, those who were better at emotional regulation showed less aggressive behavior than those with worse emotional regulation. For individuals with better inhibitory control, however, emotional regulation had no significant effect on aggressive behavior.

Emotion regulation is a process that involves relatively higher order brain functions, such as cognitive reappraisal, future planning, and anticipation of behavioral consequences. While individuals may experience difficulty transitioning out of negative emotions, through learning, experience, and practice, the ability to regulate negative emotions can be acquired and strengthened. This cognitively based ability, if well practiced, may also modulate less cognitively based, physiological states or reactions. For example, mindfulness meditation has been found to affect autonomic activities, such as blood pressure, heart rate, and heart rate variability [36, 37]. In addition, emotion regulation may moderate the effects of less cognitively based factors on aggressive behavior. For example, Castro, Bosch [38] reported that emotion regulation exercises reduced aggressive behavior, but only for children with severe behavior problems, and not for the normal control group. Results from the current study showed that emotion regulation moderated the effects of inhibitory control on aggressive behavior and significantly benefited individuals with low inhibitory control.

Also, as previously described, aggressive behavior has been found to vary across cultures [29–31]. Results from the current study did not show significant interactive effects for increases in punishment between Blocks 1 and 2, despite of significant increases in punishment in each block (i.e., Block 1 to 2, and Block 2 to 3), corresponding to increases in provocation. Interactive effects were only significant when the provocation level was increased from medium (Block 2) to high (Block 3). One potential explanation is cultural differences. As eastern culture emphasizes social harmony or avoidance of open conflict, they have been found to report less aggression and register lower on measures of aggressive behavior (e.g.,[29]). It is possible that participants from an eastern cultural are more tolerant of lower level provocation, but would become reactive only to higher level provocation.

### Limitations

There are several limitations to the current research. First, this study used an undergraduate sample. Future research may replicate and extend this research to include different age and demographic groups. Also, this study didn’t include other potentially relevant variables, such as retaliatory tendencies. Finally, the current study examined aggressive behavior in a setting when individuals were alone. Future research may include social factors in predicting aggressive behavior. Group dynamic, social desirability or social competition may serve to modulate the associations among our main study variables.

### Conclusions

Inhibitory control is a determinant in the execution of aggressive behavior. It appears that emotion regulation may be an asset, particularly for individuals with inhibitory lower control, in modulating the effects of inhibitory control on aggression. These findings have implications for allocating training resources and refining or targeting rehabilitation programs aimed at reducing aggressive behavior in adults.
